# Nanomaterials functionalized acidic ionic organosilica as highly active catalyst in the selective synthesis of benzimidazole via dehydrogenative coupling of diamines and alcohols

**DOI:** 10.1038/s41598-024-63040-9

**Published:** 2024-05-29

**Authors:** Fatemeh Rajabi, Afsaneh Feiz

**Affiliations:** 1 Department of Science, Payame Noor University, P. O. Box: 19395-4697, Tehran, 19569 Iran; 2 R&D Center, Rahkaran Shimi Mandegar Research and Scientific Company, Karaj, Iran

**Keywords:** Tungstate-based nanocatalyst, Benzimidazole, Iionic liquid, Organosilica, Dehydrogenative coupling, Chemistry, Catalysis, Environmental chemistry, Green chemistry, Materials chemistry, Organic chemistry

## Abstract

An acidic tungstate-based zwitterionic organosilica drived simple self-condensation of tungstic acid and zwitterionic organosilane (PMO-IL-WO_4_^2−^), was remarkably demonstrated to be highly efficient and environmentally friendly catalyst for directly selective synthesis of benzimidazoles from benzyl alcohols under atmpshpheric air pressure and without any additional oxidant. The one-pot synthesis of benzimidazoles from benzyl alcohols and 1,2-phenylenediamine was efficiently achieved via direct dehydrogenative reaction using a low amount of recoverable PMO-IL-WO_4_^2−^ nanocatalyst in water under ambient conditions with a conversion efficiency of more than 90%. Enhancements in yield and selectivity of benzimidazole formation were observed when water was utilized as the solvent. Furthermore, the PMO-IL-WO_4_^2−^ nanocatalyst exhibited exceptional stability, demonstrating the ability to be effortlessly separated and reused for at least eight reaction cycles without any noticeable loss in activity or product selectivity. This method supports an eco-friendly atom economy and provides a sustainable approach to accessing benzimidazoles directly from benzyl alcohols under mild conditions, demonstrating its potential for practical applications in organic synthesis.

## Introduction

**Figure 1:**
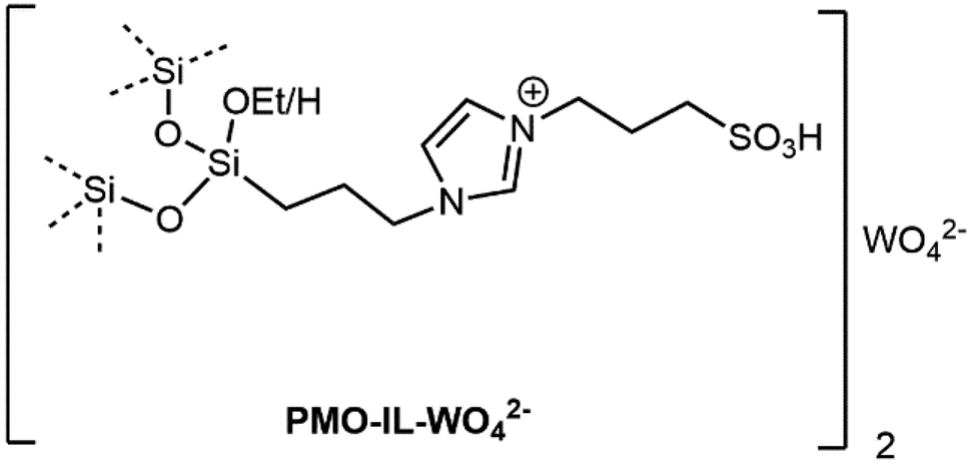
Chemical structure of PMO-IL-WO_4_^2−^ nanomaterial.

Benzimidazoles and their derivatives are crucial building blocks due to their presence in bioactive natural products and their role in the pharmaceutical industry. These compounds are an important heterocyclic unit in various drugs, such as anticancer, antiviral, antiulcer, antihistaminic, anti-hypertensive, antibacterial, anti-HIV, antifungal, anthelmintic, and antipsychotic agents^1–5^. Also they are an active structure in medicines that use for treating coronary heart disease, and Alzheimer^6^. Due to the importance of this structure there are several synthetic approaches for their preparation including condensation of 1,2-diaminobenzene with carbonyl derivatives under harsh reaction conditions^7,8^ and the coupling reaction of 1,2-di-aminobenzene with concentrated formic acid^9,10^, and direct dehydrogenative synthesis from primary alcohols^11^. Although many publications have used these procedures, researchers continue to seek alternative strategies for the synthesis of benzimidazoles due to their drawbacks. These drawbacks include harsh reaction conditions, strong acidic or alkaline conditions, stoichiometric oxidants, the use of expensive reagents, poor yields, the use of toxic solvents, severe side reactions, high reaction temperature, prolonged reaction time, and low atomic efficiency^12–16^. Therefore, the development of an efficient and environmentally friendly method for the synthesis of benzimidazoles with mild reaction conditions is a significant goal, as current procedures often result in stoichiometric salt waste and require harsh reaction conditions, strong acids or bases, and expensive reagents. To address these issues, researchers have explored various catalytic methods for the synthesis of benzimidazoles, involving both homogeneous and heterogeneous catalysts and different reaction solvents^17–20^. Heterogeneous catalysts offer advantages over homogeneous catalysts, including easier recovery and separation, as well as a reduction in the production of environmental pollution and waste. The usage of heterogeneous catalysts has been increasingly considered due to their advantages over homogeneous catalysts, including easy recovery and separation, reduced economic cost, and decreased environmental pollution and corrosion. These benefits make them an attractive option for the catalytic synthesis of benzimidazoles^21–23^. Tungsten-based catalysts have recently received considerable attention due to their wide range of environmental applications. Tungsten oxides have shown to exhibit suitable surface reactions and catalytic properties, including acidic properties, oxidation reactions, redox and adsorption properties (due to the presence of oxygen vacancies), and a photo-stimulated response under visible light. These properties make tungsten-based catalysts an attractive option for various catalytic applications. (2.6–2.8 eV band gap)^24–26^. However, because of the different solubility of tungstate anion and organic materials a phase transfer catalyst is necessary for these organic reactions. Organic salts that are liquid below 100˚C, known as Ionic Liquids (ILs), have been identified as green solvents due to their unique characteristics such as thermal and chemical stability, non-volatility, recyclability, nonexplosion and non-flammability^27–29^. Although ILs were primarily used as solvents, they have now found applications in a variety of fields such as catalysis, electrochemistry, spectroscopy, chemical separation, solid support, and material science^30,31^. In recent years, Tungstate ionic liquids have also been studied and found to be effective catalysts for various organic transformations such as CO_2_ fixation, oxidation of sulfides and alcohols, and synthesis of benzoxazoles and benzothiazoles, offering high selectivity and efficiency^32–38^. In term of sustainable synthesis and in continuation of our research efforts^39–41^ in development of green protocol for organic reactions, this contribution discloses a simple, affordable, environmentally-friendly, and gentle catalytic method for the selective synthesis of benzimidazoles in water using a previously reported PMO-IL-WO_4_^2−^ nanocatalyst^23^ (Fig. 1). By employing the innovative PMO-IL-WO_4_^2−^ nanocatalyst, this process not only minimizes environmental impact but also maximizes resource efficiency through the reusability of the catalyst.

## Experimental

### Reagents and analysis

The chemicals used in this work were obtained from suppliers and were used without purification. NMR spectra in solution were recorded by using a Bruker DPX 300 and 400 spectrometer. Elemental analyses were performed with an Elementar Vario Micro Cube (Elementar Analysensysteme GmbH, Langenselbold, Germany). An air condenser (FindenserTM, SUPER air condenser, Radleys) was employed in the catalytic reactions instead of a water condenser. The infrared spectrum was recorded using with a Shimadzu 4300 spectrometer (Shimadzu, Kyoto, Japan).

### Synthesis of N-(3-propyltriethoxysilane) imidazole

A suspension of NaH (1.45 g, 60 mmol) in a 30 mL dry tetrahydrofuran (THF) in a three-neck flask was cooled down to 0 ◦C under a nitrogen atmosphere. A solution of imidazole (4.1 g, 60 mmol) in 20 mL THF was added dropwise during 2 h. After the complete addition, the ice bath was removed and the mixture was stirred 2 h at room temperature. 3 chloropropyl trimethoxysilane (15.65 g, 65 mmol) was then added to the mixture followed by reflux overnight. The solvent was removed under vacuum and then 50 mL dichloromethane were added. The precipitate was filtered under nitrogen atmosphere. The product was then separated by distillation at 125 ◦C under vacuum. ^1^H NMR (400 MHz, CDCl_3_): δ 0.55 (t, 2 H), 1.17 (t, J = 7.5 Hz, 9 H), 1.86 (m, 2 H), 3.53 (q, J = 7.4 Hz, 6 H), 3.90 (m, 4 H), 6.89(s, 1 H), 7.02 (s, 1 H), and 7.45(s, 1 H). ^13^CNMR (100 MHz, CDCl_3_): δ 6.3, 18.9, 24.8, 49.2, 50.7, 118.8, 129.5, and 137.3.

### -(1-(3-(triethoxysilyl)propyl)-1H-imidazol-3-ium-3-yl)propane-1- sulfonate

N-(3-propyltriethoxysilane)imidazole (10 mmol, 2.73 g) was dissolved in 10 mL dry acetonitrile and was placed in ice- water bath. While it was stirred vigorously, 1,3-propane sulfone (10 mmol, 1.22 g) was added dropwise to the solution. After 30 min of stirring, the ice water bath was removed and the reaction continued under reflux for 8 h. Evaporation of the solvent under reduced pressure resulted in a white solid washing with diethyl ether, while drying under vacuum resulted in quantitative yield of 3-(1-(3-(triethoxysilyl)propyl)-1H-imidazol-3-ium- 3-yl)propane-1-sulfonate. ^1^H NMR (400 MHz, CDCl_3_): δ = 9.93 (s, 1H, NCHN), 7.70 (s, 1 H), 7.67 (s, 1 H), 4.01–3.80 (m, 4H, NCH_2_), 3.78 (q, J = 7.5 Hz, 6H, OCH_2_), 2.86 (t, J = 7.5 Hz, 2H, SCH2), 2.15 (m, 2H, CH2), 1.72 (m, 2H, CH_2_), 1.23 (t, J = 7.5 Hz, 9H, OCH_2_CH_3_), 0.57. ^13^C NMR (100 MHz, CDCl_3_): δ = 134.1, 123.6, 120.3, 59.2, 51.1, 48.6, 47.1, 21.8, 19.0, 7.9.

### Synthesis of PMO-IL-WO_4_^2−^ nanomaterials

3-(1- (3triethoxysilyl)propyl)-1H-imidazol-3-ium-3-yl)propane-1- sulfonate (10 mmol, 3.95 g) was added to 10 mL of dry acetonitrile, then tungstic acid (H_2_WO_4_) (5 mmol 1.25 g) was added to the solution under vigorous stirring at room temperature for 3 h. Fast hydrolysis and self-condensation of organo-siloxane moiety leads to the formation of acidic ionic liquid silica network with inorganic tungstate anions in the material. The resulted yellow solid was filtered and washed with acetonitrile and dried under vacuum. Elemental analysis: found C: 26.95%, N: 6.35%, S: 7.40% and H: 4.55%. ^29^Si CP-MAS NMR (99 MHz) δ -61.05 (T^1^, R–Si(OSi)_1_(OR)_2_), -70.47 (T^2^, R–Si(OSi)_2_(OR)_1_), -79.56 (T^3^, R–Si(OSi)_3_). ^13^C CP-MAS NMR (126 MHz) δ 140.81, 124.07, 49.51.

### General procedure for synthesis of benzimidazole using PMO-IL-WO_4_^2−^ nanocatalyst

In a typical reaction, a mixture containing benzyl alcohol (10 mmol) and 1,2-phenylenediamine (10 mmol), PMO-IL-WO_4_^2−^ nanocatalyst (0.01 g), and water (5 mL) was stirred at 80 °C for 4 h. After completion of reaction (TLC), the reaction mixture was filtrated off. Then ethyl acetate (10 mL) was added to the filtrate and heterogeneous PMO-IL- WO_4_^2−^ nanocatalyst was washed and recovered for the next reaction run. The combined aqueous filtrate and ethylacetate washing was separated and organic layer was dried over sodium sulfate. The solvent was removed under vacuum. The obtained solid was purified by recrystallization in ethanol to afford pure products. All synthesized compounds were characterized by ^1^H& ^13^C NMR.

## Results and discussion

Recently, tungstate ion trapped in hydrophilic/ hydrophobic nanomaterials functionalized bronsted acidic ionic liquid (PMO-IL-WO_4_^2−^) has been prepared and characterized completely in our previous reports^23^. We reported findings on the catalytic performance of the PMO-IL-WO_4_^2–^ hybrid material in the selective aerobic oxidation of primary alcohols to aldehydes using air at atmospheric pressure in water. The material's surface, which is ionic liquid-based and contain hydrophilic sulfonic acid and tungstate groups, exhibits a synergistic effect that enhances the catalyst's ability to produce aldehydes selectively in water. Based on the findings from this research, we hypothesize that PMO-IL-WO_4_^2–^ catalyst exhibits sufficient catalytic efficiency to the one-pot, direct aerobic oxidative condensation of benzyl alcohol with 1,2-phenylenediamine, resulting in the formation of 2-phenylbenzimidazole. Therefore, to show versatility and efficiency of the PMO-IL-WO_4_^2–^ nanocatalyst, we decided to test the catalyst's performance in the selective and direct synthesis of benzimidazole from benzyl alcohols, specifically using water as the solvent. The coupling reaction of benzyl alcohol and 1,2-phenylenediamine was selected as the model reaction and was carried out in different solvents and the results were summarized in Table 1. We examined the effectiveness of the catalyst in promoting the model reaction by various protic and aprotic solvents and conducting the reaction under reflux conditions. First of all, the synthesis of 2-phenylbenzimidazole from benzyl alcohol and 1,2-phenylenediamine was conducted without the catalyst, the reaction progressed at a sluggish pace, resulting in a low yield of only 8% of 2-phenylbenzimidazole after 6 hours of reaction time in boiling water. Next, the reaction was tested in aprotic solvent such as acetonitrile in the presence of 20 mg of the catalyst under reflux condition. After 6 hours, the progress of the reaction was very slow (Table 1, entry 1). The yield of the reaction also in the presence of chloroform and dichloromethane was negligible (Table 1, entry 3, 4). The efficiency of the catalyst for the model reaction was investigated in the presence of some protic solvents under reflux conditions. As a result, we observed an enhancement in the yield and selectivity, with the highest achievable yield obtained when using water as the solvent (Table 1, entry 2, 5, 6). The same reaction was carried out in toluene and solvent free conditions and it provided only moderate conversion (Table 1, entry 7, 9). Table 1 shows that in the absence of the catalysts, the product has been obtained as a trace in reaction time of 6 h (Table 1, entry 8). Further optimizations for temperature, amount of catalyst and reaction time were investigated. The yield benzimidazole was improved with increasing the reaction temperature from 70 °C to 80 °C (Table 1, entry 10, 11). Therefore, the optimum temperature for the model reaction appears to be 80 °C. The coupling reaction of benzyl alcohol and 1,2-phenylenediamine in water at 80 °C was carried out in different amount of catalyst, 10 mg, and in less reaction time (Table 1, entry 12–14). To show the important role of tungstate ions on the ionic support surface and the cooperative effect of imidazolium ionic liquid framework, sulfonic acid, and tungstate ion, the catalytic model reaction was studied in the presence of homogeneous tungstic acid, ionic support surface, neat sulfonated phenylene-bridge and in combination with tungstic acid. However desired benzimidazole was achieved in low yield (Table 1, entry 15-18). Additionally, we conducted an investigation into the cooperative effect of an imidazolium-based organosilica network and tungstate ion. We performed the oxidative coupling reaction using phenylene-bridged sulfonic acid (PMO-SO_3_H), which was prepared in our previous work, along with tungstic acid (Table 1, entry 17 and 18). However, the results of both reactions were not satisfactory. These findings suggest that both the tungstate ion and sulfonic acid play a crucial role in the ionic support and have a synergistic effect on the efficient oxidative coupling reaction of benzyl alcohol and 1,2-phenylenediamine.

**Table 1:** Optimization of catalytic conditions for dehydrogenative condensation benzyl alcohol and 1,2-phenylenediamine.


Entry	Catalyst (mg)	Solvent	T (°C )	T (h)	Yield(%)^a^
1	PMO-IL-WO_4_^2−^ (20)	MeCN	80	6	35
2	PMO-IL-WO_4_^2−^ (20)	EtOH	80	6	74
3	PMO-IL-WO_4_^2−^ (20)	CHCl_3_	80	6	31
4	PMO-IL-WO_4_^2−^ (20)	CH_2_Cl_2_	40	6	23
5	PMO-IL-WO_4_^2−^ (20)	MeOH	65	6	62
6	PMO-IL-WO_4_^2−^ (20)	H_2_O	100	6	97
7	PMO-IL-WO_4_^2−^ (20)	Toluene	115	6	60
8	-	H_2_O	80	6	trace
9	PMO-IL-WO_4_^2−^ (20)	-	100	6	64
10	PMO-IL-WO_4_^2−^ (20)	H_2_O	80	6	97
11	PMO-IL-WO_4_^2−^ (20)	H_2_O	70	6	80
12	PMO-IL-W O_4_^2−^ (10)	H_2_O	80	6	97
13	PMO-IL-W O_4_^2−^ (10)	H_2_O	80	4	97
14	PMO-IL-W O_4_^2−^ (10)	H_2_O	80	3	91
15	H_2_WO_4_ (10)	H_2_O	80	4	51
16	IL-SO_3_^−^ (10)	H_2_O	80	4	15
17	HSO_3_-PhPMO (10)	H_2_O	80	4	20
18	(HSO_3_-PhPMO + H_2_WO_4_) (10)	H_2_O	80	4	60

^a^Reaction conditions: benzyl alcohol (10 mmol), 1,2-phenylenediamine(10 mmol), solvent (3 mL)

^b^Isolated yield.

With the optimized reaction conditions the efficiency of the catalyst were evaluated by investigating variety of benzyl alcohols in dehydrogenative synthesis of benzimidazoles. The results are summarized in Table 2 and shown that all benzylic alcohol with both electrons-withdrawing and electron-donating groups gave excellent conversions.

**Table 2:**
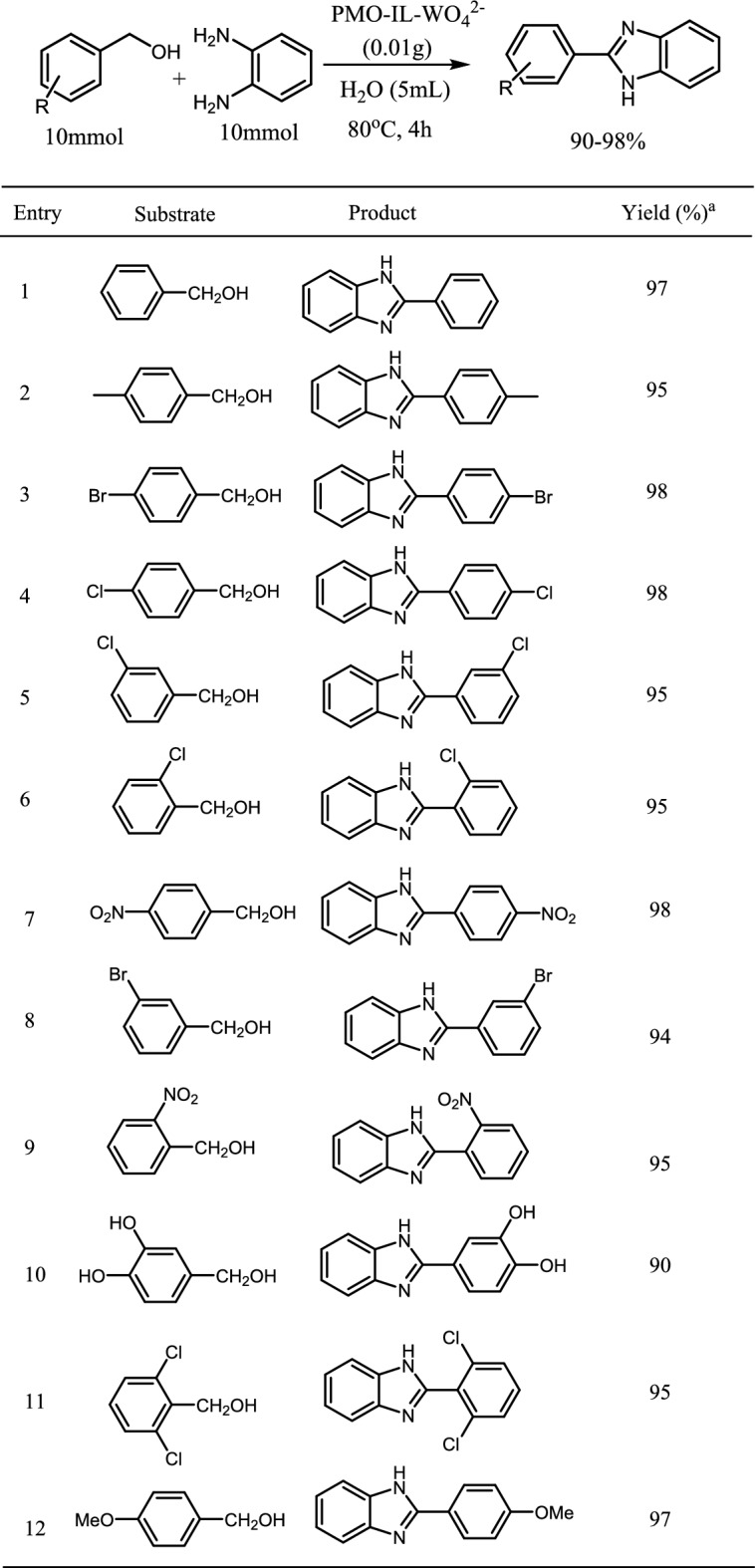
Dehydrogenative synthesis of benzimidazoles form benzyl alcohol derivatives

^a^Isolated yield.

In order to examine the impact of catalyst and leaching of active components from the support, a hot filtration test was conducted while carrying out the aerobic oxidative condensation of benzyl alcohol with 1,2-phenylenediamine under optimized conditions. The catalyst was filtered out after 3 h, and the resulting mixture was allowed to continue reacting for an additional 6 h after catalyst removal. There no progress was observed and the yield of reaction did not change (determined using GC analysis), indicating that no active catalytic species remained in the filtrate. These results confirm that the strong interaction of tungstate and imidazolium with sulfonic acid in the organosilica framework not only prevent leaching of active species, but also boost the catalytic performance of the catalyst through combined and synergic effects.

**Figure 2:**
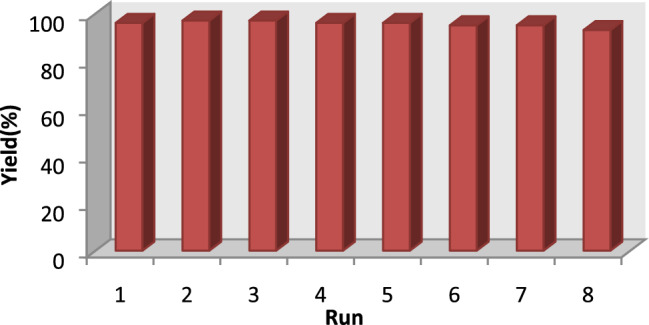
Reusability of PMO-IL-WO_4_^2−^ catalyst in dehydrogenative synthesis of benzimidazoles form benzyl alcohol.

After achieving success with the catalyst, in the next step, our subsequent research focused on examining its potential for reuse in model reaction. The PMO-IL-WO_4_^2−^ nanocatalyst can be reused 8 times without any loss of activity (Fig. 2).

Table 3 compares the efficiency and capability of PMO-IL-WO_4_^−2^ catalyst in the synthesis of benzimidazoles with some other heterogeneous catalytic systems. Table 3 shows that all catalysts have good activity in the synthesis of benzimidazoles, but some of these methods suffer from one or more of the drawbacks such as using an organic solvent, base, higher temperatures and require longer reaction times to afforded reasonable yields. The present catalytic system offered high catalytic activity under mild and green reaction conditions without any additives or organic solvent for the synthesis of benzimidazole.

**Table 3:** Comparison of catalytic activities in dehydrogenative synthesis of benzimidazole from benzyl alcohol.

Entry	Catalyst (mg)	Solvent/additive	T (°C )	T (h)	Yield(%)^a^	References
1	Ir/TiO_2_	Mesitylene, Ar	120	18	97	^11^
2	Pd-NPs/Cu-MOF	Solvent free, air, Na_2_CO_3_	120	12	88	^18^
3	Co-PNNH pincer	dry toluene, 4 Å MS, NaHBEt_3_	150	24	99	^14^
4	Ru(OH)x/TiO_2_	toluene, O_2_, 60 min/ 1,4-dioxane, air	80	2	90	^42^
5	PMO-IL-WO_4_^2−^ (10)	H_2_O, air	80	4	97	This work

**Scheme 1:**
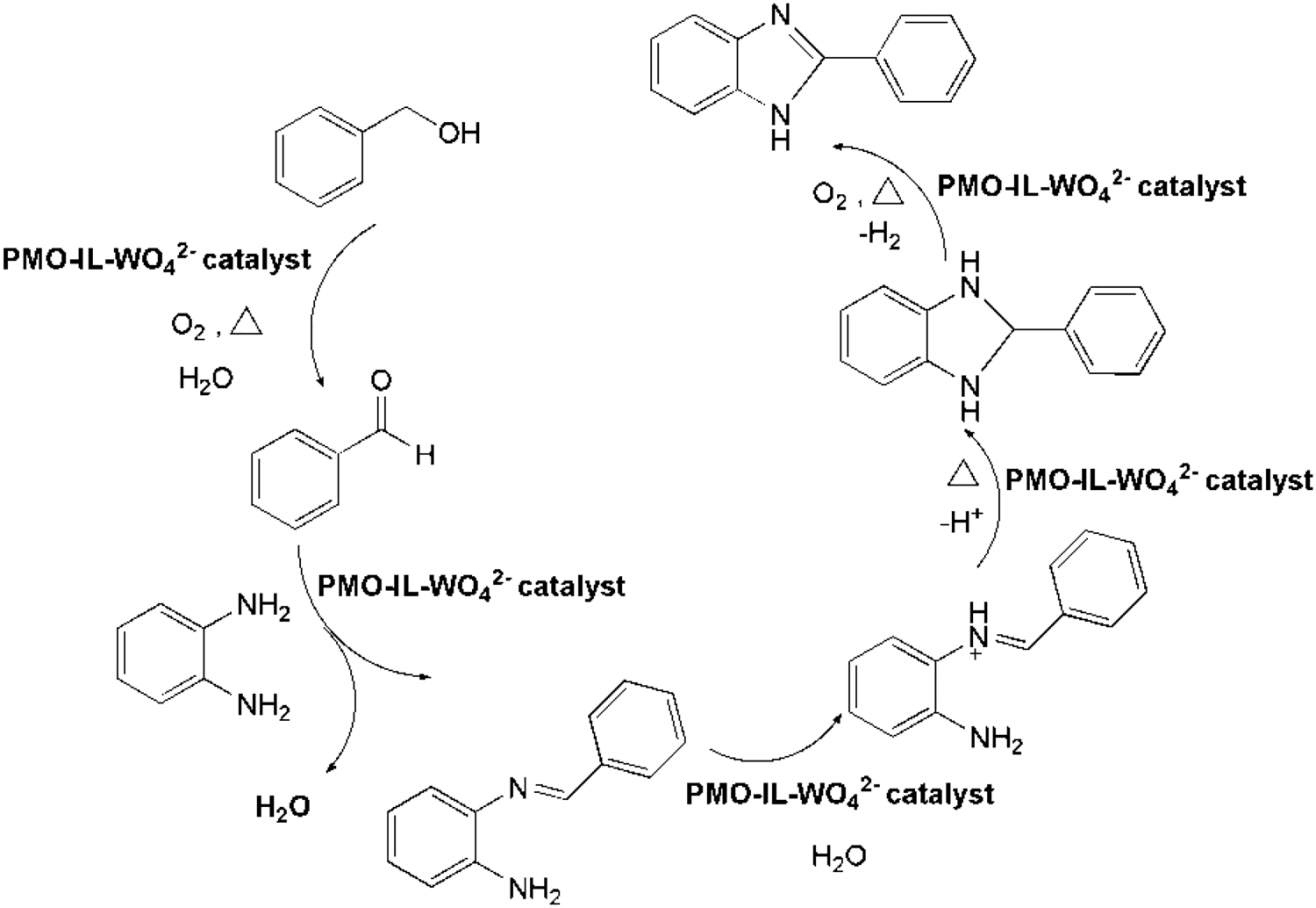
Proposed mechanism for dehydrogenative synthesis of benzimidazole from benzyl alcohol using PMO-IL-WO_4_^2−^ nanocatalyst.

A proposed reaction pathway for a sustainable and eco-friendly process for the synthesis of benzimidazole is illustrated in Scheme 1. Initially, benzyl alcohol undergoes aerobic oxidation in the presence of PMO-IL-WO_4_^−2^ catalyst to yield benzaldehyde. The generated benzaldehyde then condensed with phenylenediamine to form an imine intermediate along with the liberation of a water molecule. This imine intermediate converts to corresponding dihydrobenzimidazole which can undergo catalytic dehydrogenation in the presence of PMO-IL-WO_4_^−2^ nanocatalyst under atmospheric air pressure. This process leads to the formation of the final benzimidazole product.

## Conclusions

In conclusion, tungstate-based ionic liquid hybrid material (PMO-IL-WO_4_^2−^) has been found to be a highly efficient catalyst for the synthesis of benzimidazole using aerobic dehydrogenative coupling of 1,2-diamines and alcohols under mild and green reaction conditions. Moreover, the catalyst is heterogeneous in nature and could be easily separated and recovered at least eight times without loss of activity that makes it suitable for practical applications. By conducting the reaction under mild conditions, we aimed to assess the catalyst's effectiveness and its potential for promoting this particular benzimidazole synthesis in the presence of atmospheric air and without any additional oxidant. This contribution presents a cutting-edge approach to sustainable synthesis of benzimidazoles by utilizing eco-friendly and reusable catalysts.

### Supplementary Information

Supplementary Information.

## Data Availability

The data that support the findings of this study are available on request from corresponding author.
