# Supplementary material for: Nanomaterials functionalized acidic ionic organosilica as highly active catalyst in the selective synthesis of benzimidazole via dehydrogenative coupling of diamines and alcohols
Source: Sci Rep. 2024 May 29;14:12342. doi: 10.1038/s41598-024-63040-9 (PMC11137086; doi:10.1038/s41598-024-63040-9)
Supplement: Supplementary file 1 — Supplementary Information. [file 41598_2024_63040_MOESM1_ESM.docx]

Nanomaterials functionalized acidic ionic organosilica as highly active catalyst in the selective synthesis of benzimidazole via dehydrogenative coupling of diamines and alcohols

Fatemeh Rajabi ^a*^ and Afsaneh Feiz ^b^

^a^ Department of Science, Payame Noor University, P. O. Box: 19395-4697, Tehran 19569, Iran, Email: f_rajabi@pnu.ac.ir

^b^ R&D Center, Rahkaran Shimi Mandegar Research and Scientific Company, Karaj, Iran.

**NMR Data and Spectra**

**NMR Data**

2-phenyl-1H-benzo[d]imidazole: white solid. m.p. 286-290 ^o^C ^1^. ^1^H NMR (300 MHz, DMSO) δ 12.96 (s, 1H), 8.20 (d, *J* = 7.5 Hz, 2H), 7.74 (d, *J* = 7.5 Hz, 1H), 7.58 – 7.42 (m, 4H), 7.22-7.10 (m, 2H); ^13^C NMR (75 MHz, DMSO) δ 151.5, 143.1, 135.2, 130.5, 130.1, 129.4, 126.8, 122.7, 122.1, 119.8, 112.2.

2-(2-nitrophenyl)-1H-benzo[d]imidazole: cream solid. m.p. 210-212 ^o^C ^1^. ^1^H NMR (300 MHz, DMSO) δ 13.15 (s, 1H), 8.81 – 8.32 (m, 4H), 7.72 (d, *J* = 8.5 Hz, 2H), 7.25 (s, 2H); ^13^C NMR (75 MHz, DMSO) δ 149.1, 147.8, 143.9, 135.9, 132.9, 131.8, 131.5, 127.2, 123.9, 122.8, 121.9, 119.1, 112.6.

2-(4-methylphenyl)-1H-benzo[d]imidazole: white solid. m.p. 262-265 ^o^C ^1^. ^1^H NMR (300 MHz, DMSO) δ 12.92 (s, 1H), 8.16 (d, *J* = 7.8 Hz, 2H), 8.07 (d, *J* = 7.8 Hz, 2H), 7.75 – 7.04 (m, 7H), 7.41 (d, *J* = 7.7 Hz, 2H), 2.54 (s, 3H); ^13^C NMR (75 MHz, DMSO) δ 151.4, 143.6, 139.8, 135.1, 129.4, 127.6, 126.3, 122.4, 121.8, 118.6, 111.5, 20.8.

2-(4-chlorophenyl)-1H-benzo[d]imidazole: white solid. m.p. 289-292 ^o^C ^1^. ^1^H NMR (300 MHz, DMSO) δ 13.02 (s, 1H), 8.31 – 8.15 (m, 2H), 7.90 – 7.62 (m, 4H), 7.25 (dd, *J* = 8.6, 5.2 Hz, 2H); ^13^C NMR (75 MHz, DMSO) δ 150.3, 143.9, 134.8, 134.5, 128.9, 128.7, 122.9, 121.9, 119.0, 111.6.

2-(2-chlorophenyl)-1H-benzo[d]imidazole: light brown solid. m.p. 231-233 ^o^C ^2^. ^1^H NMR (300 MHz, DMSO) δ 12.64 (s, 1H), 8.61 – 8.52 (m, 1H), 7.82 (s, 2H), 7.64 – 7.35 (m, 5H); ^13^C NMR (75 MHz, DMSO) δ 149.8, 143.7, 135.6, 131.3, 130.7, 129.2, 128.1, 122.5, 119.6, 112.4.

2-(4-nitrophenyl)-1H-benzo[d]imidazole: light yellow solid. m.p. 314-316 ^o^C ^2^. ^1^H NMR (300 MHz, DMSO) δ 13.35 (s, 1H), 8.86 – 8.31 (m, 4H), 7.73 (d, *J* = 8.6 Hz, 2H), 7.31 (s, 2H); ^13^C NMR (75 MHz, DMSO) δ 149.3, 147.8, 143.6, 136.2, 135.3, 127.3, 124.3, 122.2, 119.4, 112.4.

2-(3-bromophenyl)-1H-benzo[d]imidazole: brown solid. m.p. 280-282 ^o^C ^1^. ^1^H NMR (300 MHz, DMSO) δ 13.07 (s, 1H), 8.25 (s, 1H), 8.18 (d, *J* = 7.5 Hz, 1H), 7.74 – 7.39 (m, 5H), 7.25 (s, 3H); ^13^C NMR (75 MHz, DMSO) δ 149.4, 143.8, 134.7, 131.9, 131.12, 128.5, 125.2, 123.5, 122.8, 122.3, 122.1, 119.4, 111.2.

2-(2,6-dichlorophenyl)-1H-benzo[d]imidazole: brown solid. m.p. 275-277 ^o^C ^2^. ^1^H NMR (300 MHz, DMSO) δ 12.91 (s, 1H), 7.84 – 7.49 (m, 5H), 7.39 – 7.12 (m, 2H); ^13^C NMR (75 MHz, DMSO) δ 147.1, 143.6, 135.4, 134.5, 132.8, 131.2, 128.5, 122.9, 121.8, 119.6, 112.2.

2-(4-carboxyphenyl)-1H-benzo[d]imidazole: white solid (1.96g, 96%). m.p. > 320 ^o^C. ^1^H NMR (300 MHz, DMSO) δ 13.08 (s, 1H), 8.40 – 8.31 (m, 2H), 8.20 (d, *J* = 8.5 Hz, 2H), 7.72 (d, *J* = 3.4 Hz, 2H), 7.25 (dd, *J* = 6.0, 3.4 Hz, 2H); ^13^C NMR (75 MHz, DMSO) δ 167.2, 150.4, 133.9, 131.8, 130.3, 129.5, 127.2, 126.8, 122.9, 122.6.

2-(3,4-dihydroxyphenyl)-1H-benzo[d]imidazole: light yellow solid. m.p. 270-273 ^o^C ^2^. ^1^H NMR (300 MHz, DMSO) δ 12.89 (s, 1H), 8.05 (d, *J* = 7.5 Hz, 2H), 7.72 – 7.31 (m, 4H), 7.12 (s, 2H); ^13^C NMR (75 MHz, DMSO) δ 149.8, 143.7, 135.2, 134.4, 130.9, 129.5, 128.9, 128.5, 128.0, 122.7, 121.8, 119.1, 111.7.

2-(4-bromophenyl)-1H-benzo[d]imidazole: white solid. m.p. 286-290 ^o^C ^1^. ^1^H NMR (300 MHz, DMSO) δ 12.88 (s, 1H), 8.02 (d, *J* = 8.3 Hz, 2H), 7.69 – 7.20 (m, 4H), 7.19 – 6.91 (m, 2H); ^13^C NMR (75 MHz, DMSO) δ 150.2, 132.2, 131.9, 131.6, 131.1, 129.5, 128.5, 123.3, 122.5, 119.9, 112.6.

**NMR Spectra**


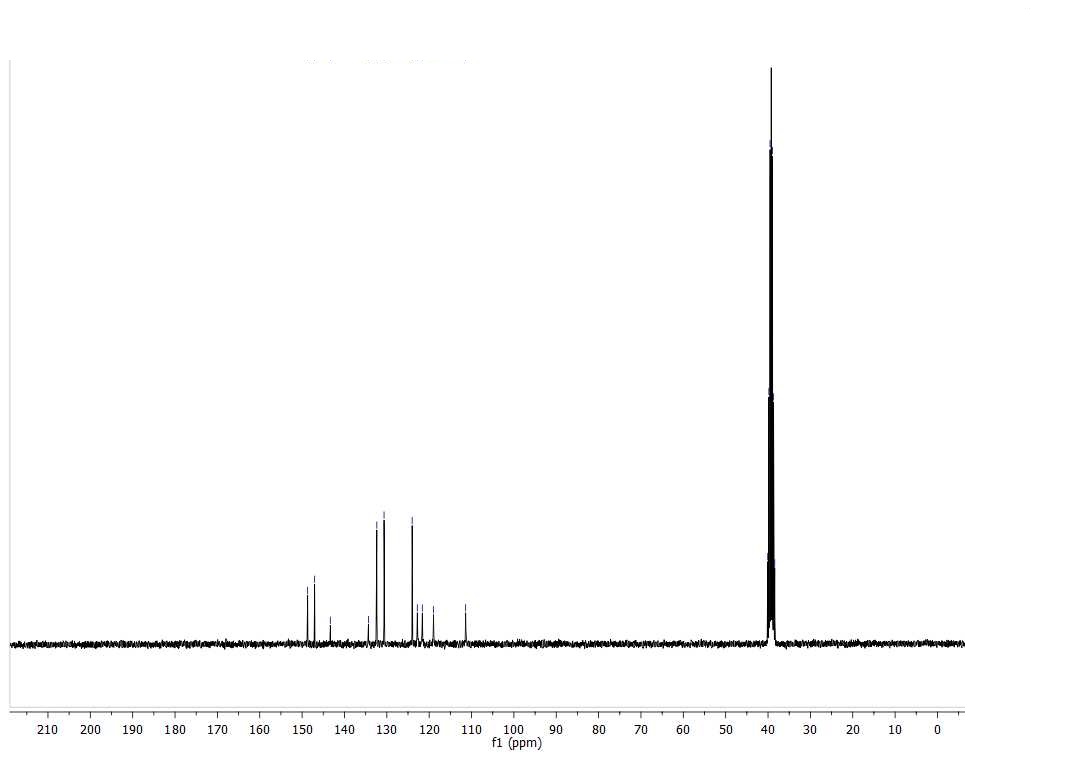


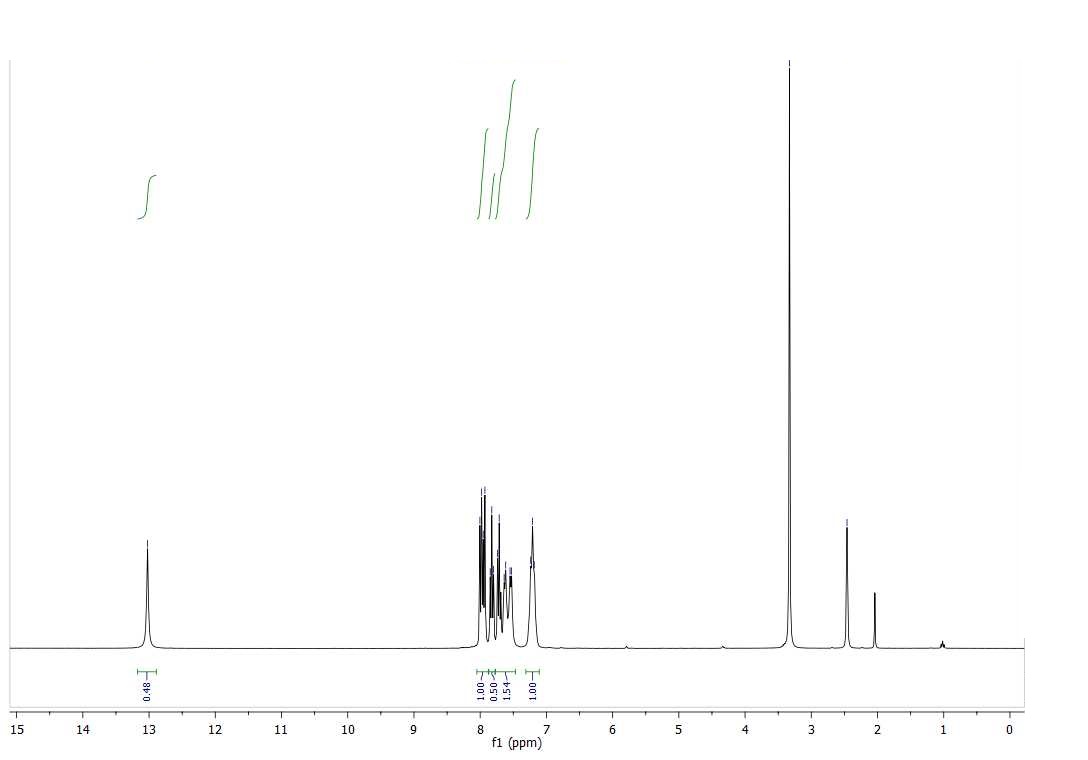


**Figure S1.** 2-(2-nitrophenyl)-1H-benzo[d]imidazole


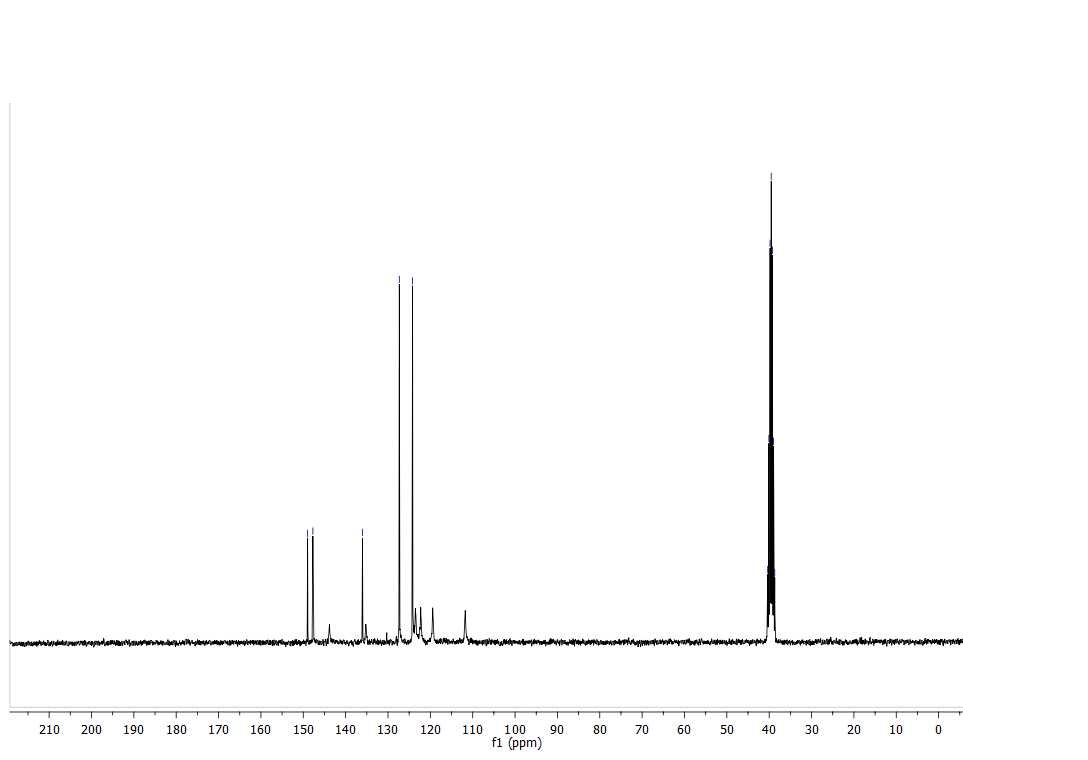

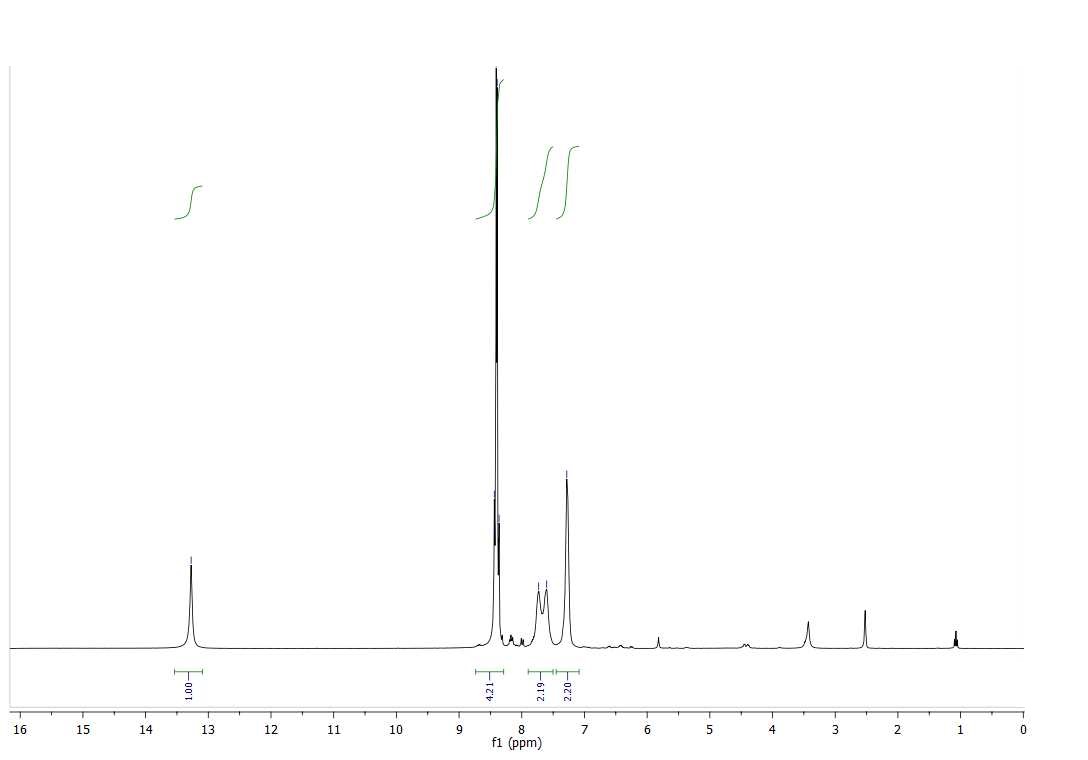


**Figure S2.** 2-(4-nitrophenyl)-1H-benzo[d]imidazole


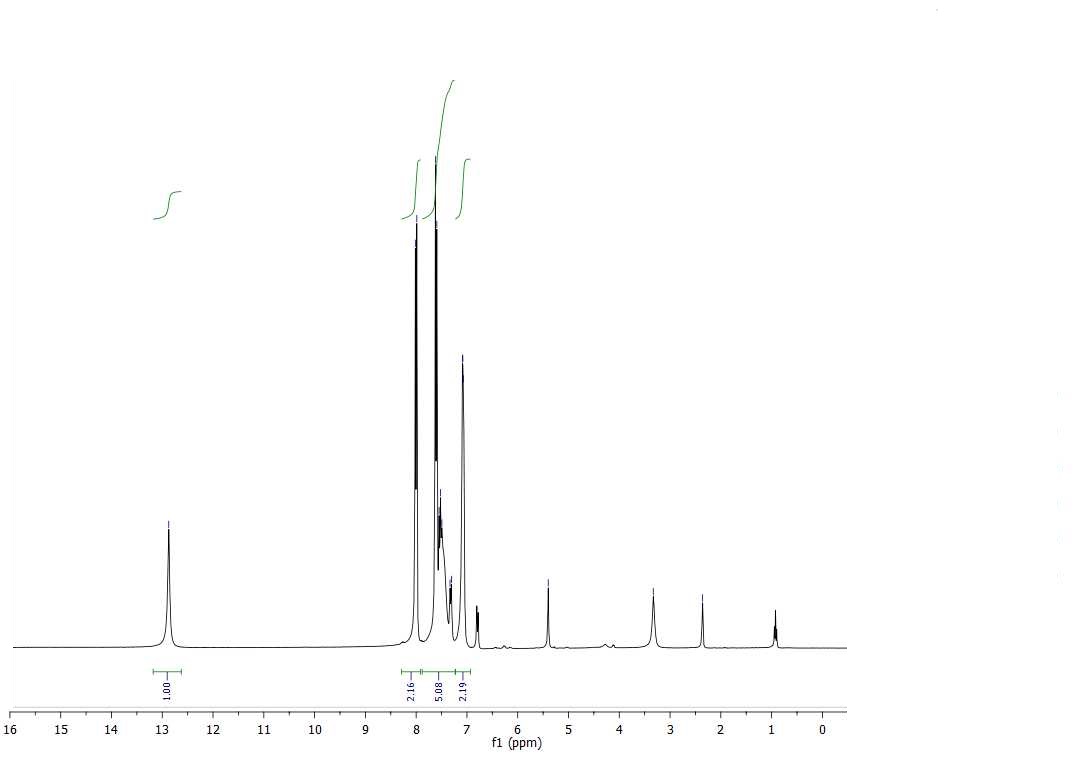

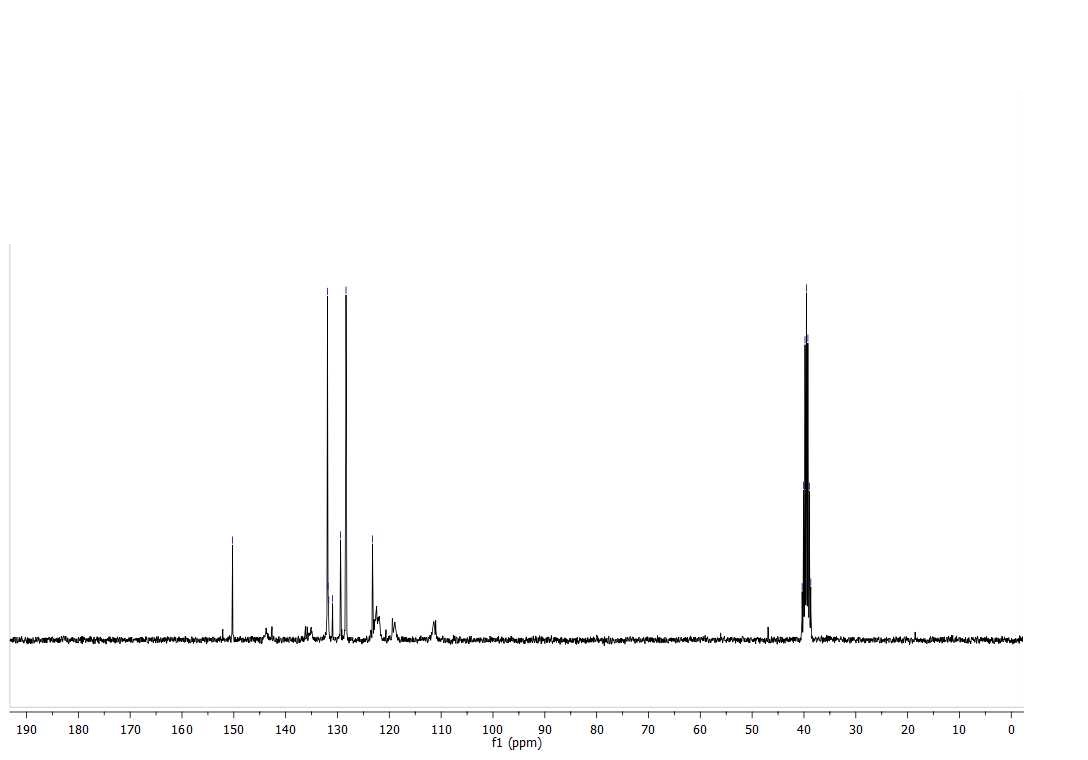


**Figure S3.** 2-(4-bromophenyl)-1H-benzo[d]imidazole


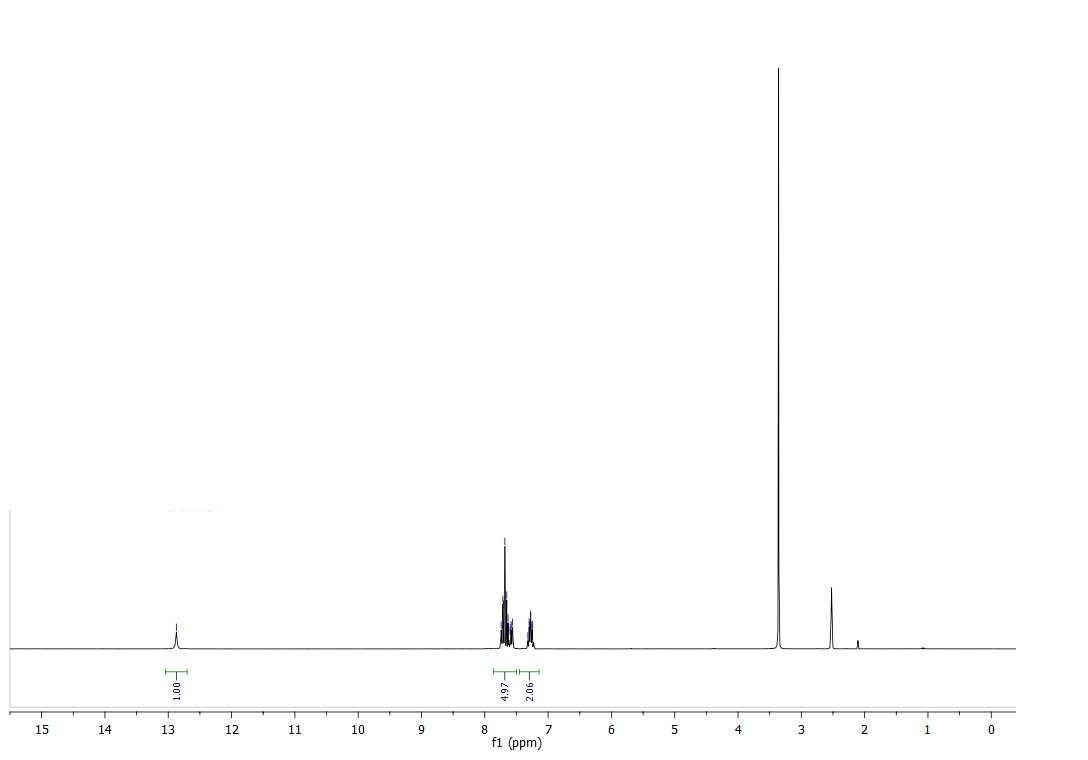


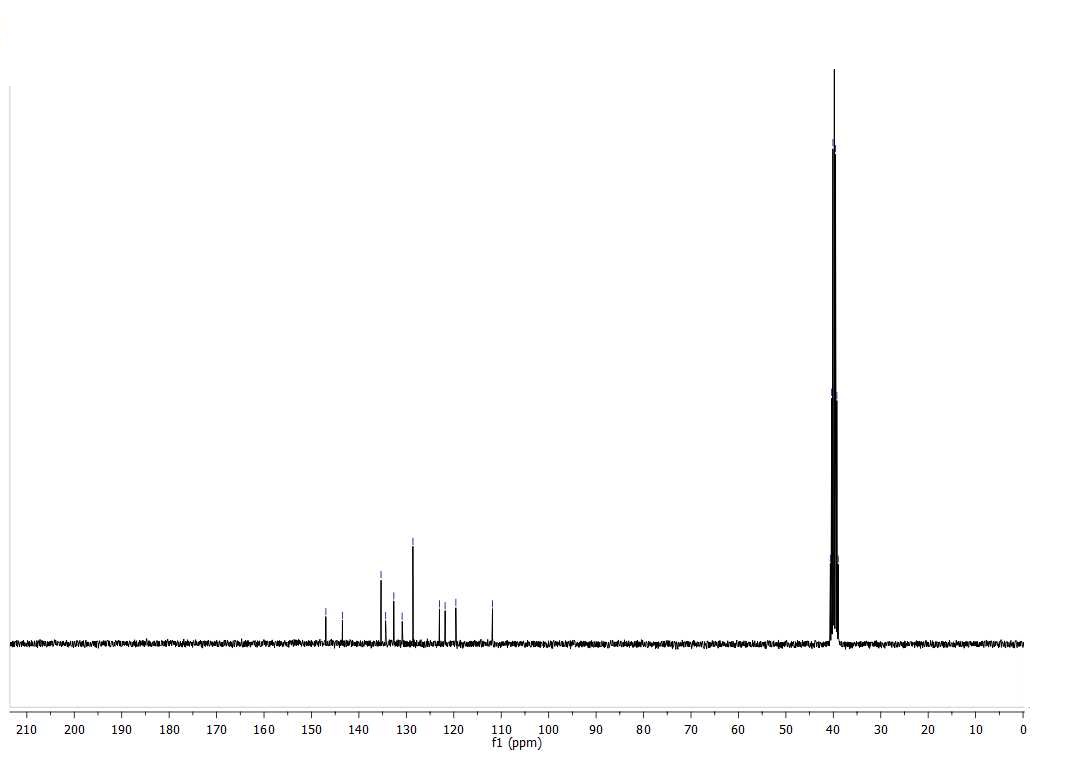


**Figure S4.** 2-(2,6-dichlorophenyl)-1H-benzo[d]imidazole


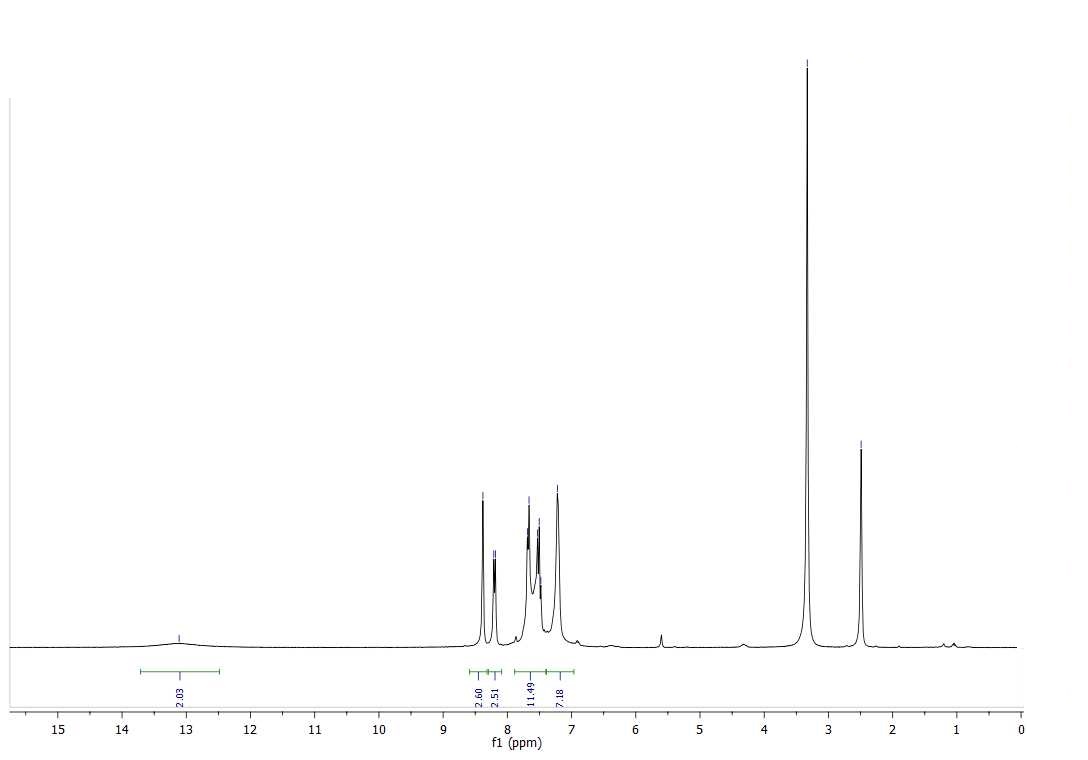


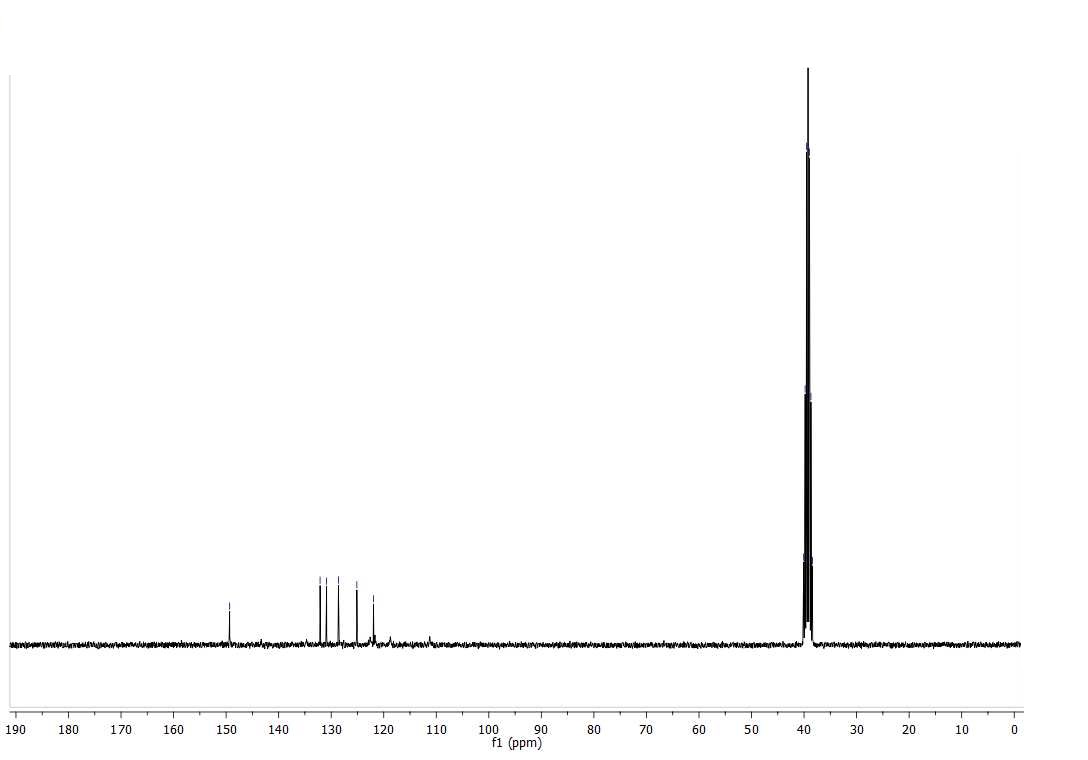


**Figure S5.** 2-(3-bromophenyl)-1H-benzo[d]imidazole

**Refrences**

1. D.Saha, A. Saha, B. C. Ranu, *Green Chem.* **2009,** *11*, 733.
2. N. H. Cano, J. G. Uranga, M. Nardi, A. Procopio, D. A. Wunderlin and A. N. Santiago, *Beilstein J. Org. Chem.* **2016,** *12,* 2410.
